# Emerging Roles of N^6^-Methyladenosine on HIV-1 RNA Metabolism and Viral Replication

**DOI:** 10.3389/fmicb.2018.00576

**Published:** 2018-03-28

**Authors:** Sebastián Riquelme-Barrios, Camila Pereira-Montecinos, Fernando Valiente-Echeverría, Ricardo Soto-Rifo

**Affiliations:** Molecular and Cellular Virology Laboratory, Virology Program, Institute of Biomedical Sciences, Faculty of Medicine, Universidad de Chile, Santiago, Chile

**Keywords:** N^6^-methyladenosine, HIV-1 genomic RNA, gene expression, Rev, YTHDF proteins

## Abstract

N^6^-methyladenosine (m^6^A) is the most abundant internal modification present in Eukaryotic mRNA. The functions of this chemical modification are mediated by m^6^A-binding proteins (m^6^A readers) and regulated by methyltransferases (m^6^A writers) and demethylases (m^6^A erasers), which together are proposed to be responsible of a new layer of post-transcriptional control of gene expression. Despite the presence of m^6^A in a retroviral genome was reported more than 40 years ago, the recent development of sequencing-based technologies allowing the mapping of m^6^A in a transcriptome-wide manner made it possible to identify the topology and dynamics of m^6^A during replication of HIV-1 as well as other viruses. As such, three independent groups recently reported the presence of m^6^A along the HIV-1 genomic RNA (gRNA) and described the impact of cellular m^6^A writers, erasers and readers on different steps of viral RNA metabolism and replication. Interestingly, while two groups reported a positive role of m^6^A at different steps of viral gene expression it was also proposed that the presence of m^6^A within the gRNA reduces viral infectivity by inducing the early degradation of the incoming viral genome. This review summarizes the recent advances in this emerging field and discusses the relevance of m^6^A during HIV-1 replication.

## Introduction

### HIV-1 Gene Expression

Human immunodeficiency virus type-1 (HIV-1) is a lentivirus belonging to the *Retroviridae* family and is the etiological agent of the acquired immunodeficiency syndrome (AIDS). HIV-1 mainly infects immune cells including T-lymphocytes, dendritic cells, macrophages and microglia. The viral replication cycle begins with the interaction between the CD4 receptor present in the target cell and the viral surface glycoprotein gp120, which leads to the consequent fusion of both membranes mediated by gp41. Once the viral capsid enters the cell, the HIV-1 genomic RNA (gRNA) is retrotranscribed into a double-stranded DNA molecule that is imported to the nucleus and integrated into a host chromosome. Transcription of the proviral DNA is commanded by the RNA polymerase II, which recognizes the promoter present within the 5′-long terminal repeat (5′-LTR) and drives the synthesis of a unique transcript of 9-kb identical to the gRNA present in the viral particle. The alternative use of splicing donors and acceptors within the 9-kb gRNA give rise to over 100 viral transcripts that ensures the expression of the nine genes present within the viral genome (Karn and Stoltzfus, 2012; Ocwieja et al., 2012). Viral transcripts are mainly classified according to their size as 2-kb (multiply spliced), 4-kb (singly spliced) and 9-kb (full-length unspliced) (Purcell and Martin, 1993). Multiply spliced mRNAs code for the regulatory proteins Tat and Rev and the accessory protein Nef and are the predominant mRNA species early during viral replication. Singly spliced mRNAs encode the surface glycoprotein Env as well as the accessory proteins Vif, Vpr, and Vpu and the full-length unspliced mRNA is used for the synthesis of the structural proteins Gag and Gag-Pol. These intron-containing mRNA species predominate later during viral replication once the viral protein Rev accumulates within the nucleus (Malim and Cullen, 1993). Upon nuclear export, viral mRNAs recruit host ribosomes in order to synthesize the different viral proteins necessary for the completion of the viral replication cycle (Karn and Stoltzfus, 2012; Rojas-Araya et al., 2015). The 9-kb gRNA plays two critical roles within the cytoplasm since it acts as the messenger RNA for Gag and Gag-Pol synthesis but also as the genome packaged into new viral particles (Kim et al., 1989; Pomerantz et al., 1990; Butsch and Boris-Lawrie, 2002). Many of the molecular mechanisms governing the post-transcriptional steps of the HIV-1 replication cycle still remain unclear. Interestingly, recent data showed that the presence of N^6^-methyladenosine (m^6^A) residues along the gRNA are important in regulating the cytoplasmic fate of viral transcripts (Kennedy et al., 2016; Lichinchi et al., 2016a; Tirumuru et al., 2016; Lu et al., unpublished). The roles of this RNA modification during viral replication have just started to be elucidated.

### Post-transcriptional Regulation by N^6^-Methyladenosine

**FIGURE 1:**
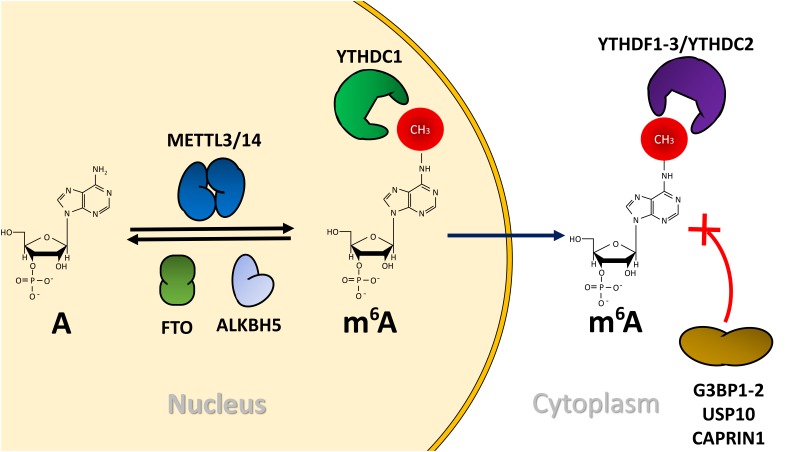
m^6^A and its associated machinery. In the nucleus, the methyltransferase complex composed by METTL3 and METTL14 (other components were omitted for simplicity) methylates adenosines present in mRNA. This methyl group can be removed by one of the RNA demethylases ALKBH5 or FTO. Methylated mRNAs are recognized by YTHDC1 in the nucleus and by YTHDF1-3 or YTHDC2 in the cytoplasm. The presence of m^6^A also repeals RNA-binding proteins such as G3BP1-2, USP10 or CAPRIN1.

Similar to proteins and DNA, mRNA undergoes chemical modifications that impact different steps of gene expression. N^6^-methyladenosine or m^6^A is the most abundant internal modification described so far in eukaryotic mRNA (Meyer and Jaffrey, 2017; Roignant and Soller, 2017). The methylated adenosine occurs mainly in the consensus motif RRACH (R = G or A; H = A, C, or U) and are mainly concentrated close to stop codons and in 5′- and 3′-unstranslated regions (Dominissini et al., 2012; Meyer et al., 2012). The methylation of adenosine residues is catalyzed by a methyltransferase complex mainly composed by an heterodimer of methyltransferase-like 3 (METTL3) and methyltransferase-like 14 (METTL14) together with the cofactor Wilms tumor 1-associated protein (WTAP) and are denominated as m^6^A “writers” (Liu et al., 2014; Ping et al., 2014) (**Figure 1**). The methyl group from m^6^A can be removed, at least *in vitro*, by two RNA demethylases, Fat mass and obesity associated protein (FTO) and α-ketoglutarate-dependent dioxygenase homolog 5 (ALKBH5), which are known as m^6^A “erasers” (Jia et al., 2011; Zheng et al., 2013) (**Figure 1**). Despite FTO has been shown to demethylate the body of certain mRNAs (Zhao et al., 2014), it was recently reported that the N^6^,2′-*O*-dimethyladenosine (m^6^Am) modification adjacent to the 7-methylguanosine cap structure rather than m^6^A is the main substrate of this demethylase (Mauer et al., 2016). Moreover, although ALKBH5 is now considered as the major m^6^A eraser of mRNA, the reversibility of the methylation process in cells has been recently challenged (Darnell et al., 2017; Ke et al., 2017; Rosa-Mercado et al., 2017).

The m^6^A modification in mRNA is specifically recognized by members of the YT521B homology (YTH) family of proteins, which together are called m^6^A “readers” (Meyer and Jaffrey, 2017; Patil et al., 2017). To date, three YTHDF (YTH domain Family) members, YTHDF1, YTHDF2, and YTHDF3 localized in the cytoplasm (Dominissini et al., 2012; Wang et al., 2014, 2015; Shi et al., 2017), and two YTHDC (YTH domain containing) proteins, YTHDC1 located in nucleus (Xu et al., 2014) and YTHDC2 located in cytoplasm (Morohashi et al., 2011) have been identified as m^6^A readers. YTHDF proteins are closely related and contain the YTH domain (responsible of m^6^A binding) at their C-terminus as well as a low complexity amino-terminal domain rich in Q, N, and P residues (Meyer and Jaffrey, 2017; Patil et al., 2017) (**Figure 1**). An interesting aspect of YTHDF proteins is that, despite their close similarity, they have been associated to different regulatory processes. As such, while binding of YTHDF1 was shown to promote enhanced translational rates of its mRNA targets (Wang et al., 2015), binding of YTHDF2 was shown to induce mRNA degradation (Wang et al., 2014). Interestingly, it was recently reported that YTHDF3 promotes the function of its two homologs, favoring translation when associated with YTHDF1 and mRNA decay when associated to YTHDF2 (Shi et al., 2017). These observations are in line with the notion that m^6^A is an important regulator of mRNA turnover (Ke et al., 2017). On the other hand, the nuclear m^6^A reader YTHDC1 was shown to promote exon inclusion by favoring the recruitment of the splicing factor SRSF3 while blocking the binding of SRSF10 (Xiao et al., 2016). In addition, the association of YTHDC1 with SRSF3 was also shown to promote nuclear export of m^6^A-containing mRNA (Roundtree et al., 2017). Although YTHDC2 was shown to enhance translational efficiency and degradation of mRNA targets related to spermatogenesis (Hsu et al., 2017), it is still unclear whether this protein plays m^6^A-dependent roles (Meyer and Jaffrey, 2017; Patil et al., 2017).

It should be mentioned that the ability to recognize and bind to m^6^A seems not to be restricted to members of the YT521B homology (YTH) family of proteins. Indeed, hnRNPA2/B1 was shown to bind m^6^A *in vitro* and *in vivo* in order to regulate pri-miRNA processing (Alarcón et al., 2015). In addition, FMRP was also shown to bind m^6^A in a sequence context-dependent manner (Edupuganti et al., 2017). More recently, IGF2BP 1, 2, and 3 were shown to bind m^6^A in order to promote stability and storage of mRNAs under normal and stress conditions (Huang et al., 2018). As it will be discussed below, the HIV-1 Rev protein was also shown to bind preferentially to m^6^A-containing viral RNA in order to promote viral gene expression (Lichinchi et al., 2016a).

In addition to the recruitment of reader proteins, the presence of m^6^A in mRNA can also regulate gene expression by interfering with the binding of regulatory proteins to methylated mRNAs. This phenomena was shown for G3BP1 and G3BP2, which are repelled by m^6^A in a specific sequence context resulting in decreased mRNA stability (Edupuganti et al., 2017).

Interestingly, m^6^A is also present in RNA from several viruses and has been proposed as a key regulator of viral replication (Brocard et al., 2017; Gokhale and Horner, 2017; Kennedy et al., 2017; Pereira-Montecinos et al., 2017).

### Role of m^6^A During the HIV-1 Replication Cycle

Forty years ago, Beemon and Keith (1977) reported for the first time the presence of m^6^A residues in a retroviral genome using Rous sarcoma virus (RSV) as a model. Later on, they suggested a role for this modification in RSV RNA processing (Kane and Beemon, 1985). More recently, high-throughput sequencing-based technologies allowing the analysis of the topology of m^6^A as well as the identification of the binding sites of the m^6^A cytoplasmic readers YTHDF1, 2, and 3 were employed to identify m^6^A sites along the HIV-1 gRNA (Kennedy et al., 2016; Lichinchi et al., 2016a; Tirumuru et al., 2016; Lu et al., unpublished). From these articles, it was clear that m^6^A and its associated machinery play a positive role in viral gene expression. However, it was also reported that cytoplasmic m^6^A readers play a negative role on the incoming viral RNA resulting in reduced infectivity and viral replication. The main observations of these four reports are presented below.

#### m^6^A-Mediated Nuclear Export of Rev-Dependent Transcripts

**FIGURE 2:**
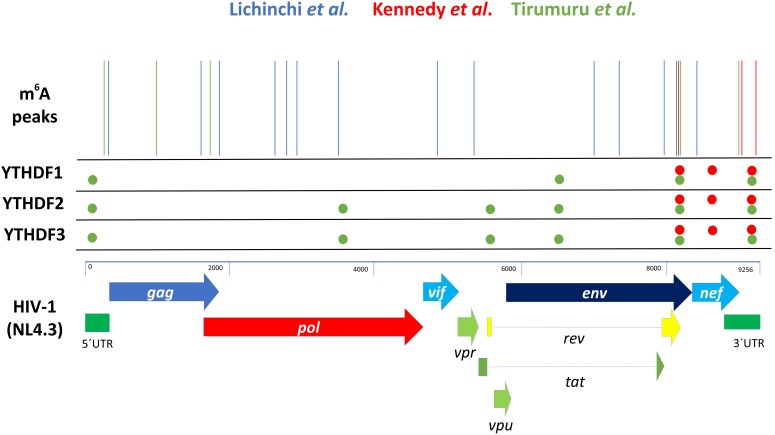
Summary of HIV-1-related m^6^A-seq and CLIP-seq data. Schematic, not to scale, representation of the position of the m^6^A peaks reported for the HIV-1 genome. The NL4.3 strain was used as a reference to compare data reported by Lichinchi et al. (2016a) (blue lines), Kennedy et al. (2016) (red lines) and Tirumuru et al. (2016) (green lines). It should be mentioned that Lichinchi et al. (2016a) used the HIV-1 LAI strain to perform m^6^A-seq. The position of YTHDF1-3 binding sites reported by Kennedy et al. (2016) (red circles) and Tirumuru et al. (2016) (green circles) are also represented.

In a first report, Lichinchi et al. (2016a) used the HIV-1 LAI strain to infect MT4 T-cells previously knocked down for the m^6^A writers METTL3 and METTL14 or the m^6^A eraser ALKBH5. The authors analyzed both total levels of gp120 mRNA by RT-qPCR and the levels of intracellular CAp24 by Western blot and used them as a surrogate of viral gene expression. Interestingly, depletion of both methyltransferases resulted in the reduction of gp120 mRNA and CAp24 protein levels indicating a positive role of m^6^A writers in viral gene expression. Consistent with this observation, depletion of the m^6^A eraser ALKBH5 resulted in a very strong (up to 10-fold) increase in the total Env mRNA levels and a milder effect on CAp24 levels. Together, these experiments showed for first time the involvement of m^6^A writers and erasers on HIV-1 gene expression. Then, authors employed the m^6^A-seq strategy (Dominissini et al., 2012; Meyer et al., 2012) and determined the topology of m^6^A during HIV-1 LAI infection. Bioinformatic analyses revealed the presence of fourteen m^6^A peaks along the HIV-1 genome mapping to different regions from the 5′-UTR to the Nef coding region (**Figure 2**). Interestingly, the authors also reported the dynamics of m^6^A in T-cells mRNAs in response to HIV-1 infection identifying 56 transcripts that are specifically methylated in infected cells. Interestingly, proteins encoded by some of these cellular mRNAs were previously linked to HIV-1 replication suggesting that m^6^A would also regulate viral replication indirectly through the post-transcriptional regulation of host transcripts.

Lichinchi et al. (2016a) put attention to a methylation peak located within the Rev Response Element (RRE) and went into characterize the molecular mechanism by which methylation of this specific region of the HIV-1 genome could impact viral gene expression and replication. Authors identified A7883 and A7877 within the stem loop IIB of the RRE as the methylated residues despite both adenosines are not present in a favorable methylation context (UG**A**CG instead of a RR**A**CH sequence context and within a stem loop structure). Since stem loop IIB is critical for Rev binding and nuclear export of Rev-dependent mRNAs (Heaphy et al., 1990), authors determined the impact of m^6^A on Rev binding to the RRE in METTL3/METTL14 or ALKBH5 knockdown 293T cells expressing HIV-1. Authors observed that depletion of METTL3/METTL14 diminished the Rev-RRE interaction while ALKBH5 depletion had the opposite effect suggesting that methylation of the viral RNA favors the binding of Rev. Finally, authors performed A to G mutations at positions 7883 and 7877 (including compensatory substitutions in order to avoid changes in the RRE folding) and evaluated total gp120 mRNA levels and relative nuclear export in METTL3/METTL14 or ALKBH5 knockdown 293T cells. Interestingly, while mutation of A7877 mildly reduced gp120 mRNA levels and had no impact on nuclear export, mutation of A7883 resulted in a strong reduction in gp120 mRNA levels and nuclear export. Although previous *in vitro* studies based on NMR and RNA foot printing using non-methylated RNAs reported the critical role of A7877 for Rev binding (Kjems et al., 1992; Battiste et al., 1996), the authors proposed that the A7877 mutation renders gp120 mRNA insensitive to the effects of METTL3/METTL14 or ALKBH5 knockdown. With all these data, Lichinchi et al. (2016a) propose a mechanism in which binding of Rev to its mRNA targets could be regulated by the activity of m^6^A writers and erasers on A7877 present within stem loop IIB of the RRE. However, it is still unclear the molecular mechanism by which the presence of m^6^A favor the binding of Rev to the RRE in cells. Since m^6^A can alter RNA structures (Liu et al., 2015, 2017; Roost et al., 2015; Spitale et al., 2015), it is possible that the presence of the modification allows the RRE to adopt the optimal conformation for Rev binding. An alternative intriguing possibility is that Rev is a viral m^6^A reader with higher affinity for methylated viral transcripts. Further work is needed to clarify the m^6^A-mediated regulation of Rev activity during HIV-1 replication.

#### Accumulation of HIV-1 Transcripts by Cytoplasmic m^6^A Readers

In a second report, Kennedy et al. (2016) analyzed the impact of cytoplasmic m^6^A readers on HIV-1 gene expression. In this study, authors first analyzed the topology of m^6^A residues along the HIV-1 NL4.3 genome using infected human CD4+ CEM-SS T-cells and the photo-crosslinking-assisted m^6^A sequencing (PA-m^6^A-seq) technology, which has an improved resolution compared to the m^6^A-seq strategy (Chen et al., 2015). They identified several m^6^A sites, which were clustered at the last 1.2-kb toward the 3′ of the 9-kb HIV-1 RNA genome (**Figure 2**). In addition, authors performed PAR CLIP-seq analysis from HIV-1-expressing 293T cells and determined the binding sites of Flag-tagged YTHDF1–3 along the gRNA. Interestingly, they observed that most but not all the binding sites of the m^6^A readers coincided with m^6^A sites. Of note, the m^6^A sites coinciding with YTHDF1–3 binding sites were located at the *env/rev* overlap, the NF-kB repeats and the R region of the 3′-LTR (**Figure 2**). Authors also mapped the binding sites of YTHDF1 and 2 in the 3′-UTR of the primary isolates BaL and JR-CSF and observed a conservation of the m^6^A clusters identified in the NL4.3 genome. However, they also identified one additional m^6^A cluster in BaL and two additional clusters in JR-CSF, indicating that m^6^A-mediated regulation might also be relevant for circulating viruses.

In a functional assay aimed at evaluating the role of methylated adenosines present within the 3′-UTR, authors noticed that these residues were important for mRNA abundance. In addition, they evaluated the impact of overexpressing YTHDF1, 2 and 3 on HIV-1 gene expression in 293T cells and observed increased levels of gag, nef, tat and rev mRNAs and Gag and Nef proteins. Finally, authors focused on the role of YTHDF2 protein during infection in CEM-SS cells, using overexpression (OE) and CRISPR/Cas9-mediated knockout (KO). Consistent with a positive role of this m^6^A reader in HIV-1 gene expression, it was shown that HIV-1 replicated better in YTHDF2-OE cells and lower in YTHDF2-KO cells producing more and less Gag and Nef proteins, respectively. In summary, Kennedy et al. (2016) confirmed that the HIV-1 RNA genome is decorated with m^6^A with clusters concentrated at the 3′-UTR and reported that YTHDF1-3 proteins are recruited to the viral RNA increasing viral mRNA abundance and protein synthesis. It is noteworthy that this study not only showed the positive effect that m^6^A residues present at the 3′-UTR exert on viral gene expression but also evidenced an unexpected redundant effect of the cytoplasmic m^6^A reader proteins on the fate of HIV-1 mRNAs.

#### Degradation of the Incoming HIV-1 RNA Genome by Cytoplasmic m^6^A Readers

A third group performed m^6^A-seq from HIV-1-infected Jurkat cells, primary CD4+ T-cells and 293T cells and corroborated the presence of m^6^A sites enriched at the 5′- and 3′-UTRs of the viral RNA and within several internal positions (Tirumuru et al., 2016) (**Figure 2**). By using CLIP-seq, they also mapped the binding sites of Flag-tagged YTHDF1-3 proteins along the HIV-1 genome in infected HeLa/CD4+ cells. In agreement with Kennedy et al., 2016 they found multiple binding sites for these m^6^A readers along the HIV-1 genome (mainly at the 5′-UTR, *env* gene, *rev* gene and the 3′-UTR), some of them overlapping with identified m^6^A sites (**Figure 2**).

The authors also went into determine the impact of m^6^A readers during infection of a Firefly luciferase-expressing HIV-1 virus (using luciferase and anti-Gag Western blot as a readout of infection) by individually overexpressing and knocking down each YTHDF protein in HeLa, Jurkat and primary CD4+ cells. However and in sharp contrast with data reported by Kennedy et al. (2016) in 293T cells, they observed that overexpression of cytoplasmic m^6^A readers resulted in reduced levels of HIV-1 infection. As expected, YTHDF1-3 knockdown had the opposite effect and resulted in increased rates of HIV-1 infection. Authors provided evidence pointing to a decrease in the levels of late reverse transcription products, which was explained by reduced genomic RNA levels in YTHDFs overexpressing cells. From these data, authors suggested that m^6^A readers bind to the incoming viral RNA inducing its degradation with the resulting inhibition of infection.

Strikingly, Tirumuru et al. (2016) also reported that m^6^A promotes viral gene expression at the post-transcriptional level as they observed that knockdown of m^6^A writers inhibits Gag synthesis from the NL4.3 provirus while knockdown of the m^6^A erasers, FTO and ALKBH5, has the opposite effect.

Together, the reports by Kennedy et al. (2016) and Tirumuru et al. (2016) suggest that the presence of m^6^A along the HIV-1 gRNA has a double function on viral RNA metabolism and the replication cycle by triggering the degradation of the genomic RNA that enters the cell but favoring its abundance and/or translation during the late stages of infection. The molecular determinants influencing the opposites effects of YTHDF1-3 proteins on the HIV-1 gRNA are still unknown.

#### Cytoplasmic m^6^A Readers Regulate HIV-1 Production and Infectivity

In an effort to gain further insights into the role of YTHDF1-3 proteins on viral replication, the Wu group confirmed that overexpression of YTHDF1-3 proteins in target HeLa cells results in a reduction of the early and late RT products due to the decline in the gRNA levels (Lu et al., unpublished). Given the fact that the firefly luciferase reporter present in the HIV-1-Luc/VSV-G virus contains m^6^A sites, they used wild type HIV-1 NL4.3 to infect HeLa/CD4+ cells overexpressing each YTHDF protein. By using this alternative approach, authors showed a reduction in Gag protein levels in cells and CAp24 in supernatants, which was also due to lower levels of early and late RT products and reduced *gag* mRNA compared with control cells. Authors also showed that YTHDF1-3 proteins binds the HIV-1 gRNA in infected HeLa/CD4+ cells thus, confirming their previous data obtained by CLIP-seq experiments (Tirumuru et al., 2016; Lu et al., unpublished).

The authors then focused on a m^6^A peak present within the 5′-UTR of the gRNA previously identified by m^6^A-seq (Tirumuru et al., 2016). According to the authors, this peak harbors two m^6^A consensus motifs (GGACU) with residue A198 located within the primer binding site (PBS) and residue A242 located at the base of SL1 as the potentially methylated adenosines (Lu et al., unpublished). Interestingly, this m^6^A peak also overlaps with a CLIP-seq peak for the three YTHDFs proteins previously reported by the authors (Tirumuru et al., 2016). *In vitro* binding assays using a fragment of the 5′-UTR and recombinant proteins confirmed the preferential binding of YTHDF1-3 proteins to a methylated RNA.

Due to the critical role of the 5′-UTR in viral replication, authors performed single and double A to G mutations at positions 198 and 242 of the NL4.3 genome and analyzed their impact on viral replication in 293T cells. Despite these mutants expressed the same levels of full-length Gag, authors observed a slight increase in processing intermediates such as CA-MA p41 and CAp24. They also observed a mild increase in the levels of CAp24 in the supernatants of cells transfected with the mutant viruses suggesting that the lack of adenosines 198 and/or 242 within the 5′-UTR favors Gag synthesis and virus production. However, infectivity assays performed in TZM-bl cells using equal amounts of CAp24 revealed a lower infectivity of mutant viruses thus, suggesting that these adenosine residues are important for infectivity.

Authors also performed YTHDF1-3 knockdown in 293T producer cells and analyzed the impact on Gag and CAp24 levels from cell lysates and CAp24 in supernatants. Interestingly, while Gag and CAp24 levels were reduced in cells and supernatants, infectivity of viruses generated from YTHDF1 or YTHDF3 knockdown cells was slightly higher when compared to the virus produced from control cells. In contrast, viruses generated from YTHDF2 knockdown cells presented a 25% reduction in infectivity compared to the controls, indicating that knockdown of YTHDF proteins in producer cells differentially impact infectivity of HIV-1. In agreement with this idea, authors showed that YTHDF1-3 proteins form a complex with Gag in an RNA-dependent manner suggesting that m^6^A cytoplasmic readers might interfere with the proper assembly of viral particles.

Together, these new data confirm the negative role of cytoplasmic m^6^A readers of target cells on the early steps of the HIV-1 replication cycle. These data also provide evidence for a negative role of methylation and cytoplasmic m^6^A readers from producer cells on viral infectivity.

#### hnRNPA1/B2 as a Potential HIV-1 m^6^A Reader

The role of the hnRNPA1/B2 protein in the post-transcriptional control of the HIV-1 genomic RNA has been studied since 2001. Different reports indicate that hnRNPA1/B2 regulates cytoplasmic trafficking of the gRNA, but not its production nor Gag synthesis (Mouland et al., 2001; Lévesque et al., 2006). In addition to this function in gRNA trafficking, a last report suggested that hnRNPA1/B2 was involved in the nuclear retention of the gRNA observed in the absence of Rev thus, having a potential role on nuclear export (Gordon et al., 2013). Although is still unclear the precise mechanism by which hnRNPA1/B2 is involved in the post-transcriptional control of the HIV-1 gRNA, a recent report proposed a novel role of this cellular protein as an m^6^A reader (Alarcón et al., 2015). However, the specific function of hnRNPA1/B2 as an m^6^A reader and the relationship of this function with its reported role on HIV-1 gRNA trafficking are completely unknown.

## Discussion

By using sequencing-based methodologies, three different groups have demonstrated that the HIV-1 genomic RNA is decorated with m^6^A (**Figure 2**). While there is a consensus in the presence of the modification at the 5′- and 3′-UTR, the presence of m^6^A at specific additional positions of the viral genome (such as the RRE) is more controversial. The most probably explanation for these differences lies in the mapping strategies used by each group. While Lichinchi et al. (2016a) and Tirumuru et al. (2016) employed the m^6^A-seq strategy that has a 100–200 nt resolution, Kennedy et al. (2016) employed the PA-m6A-seq strategy, which has an improved resolution (around 30 nt) but requires that the m^6^A site has a nearby site for 4SU incorporation (Li et al., 2016). The use of the miCLIP strategy (Linder et al., 2015), which has single-nucleotide resolution, will be instrumental in precisely map the m^6^A sites present along the HIV-1 gRNA.

Similar discrepancies arise from the binding sites of YTHDF1, 2, and 3 proteins identified by CLIP-seq analyses. While Kennedy et al. (2016) and Tirumuru et al. (2016) identified binding sites at the 3′-UTR that coincides with m^6^A peaks, Tirumuru et al. (2016) also identified YTHDFs binding sites at the 5′-UTR and coding sequences (**Figure 2**). Interestingly, while the m^6^A peak at the 5′-UTR coincides with CLIP-seq data, several binding sites for YTHDF1, 2, and 3 do not coincide with predicted m^6^A sites suggesting that cytoplasmic readers would also bind the HIV-1 genomic RNA in an m^6^A-independent manner or that the viral transcript possess additional m^6^A sites that have not been mapped yet. However, it is also possible that the different cell types used in the m^6^A-seq and CLIP-seq experiments might contribute to the differences of the m^6^A sites and YTHDFs-binding sites identified along the HIV-1 RNA genome.

There is also a consensus on the role of m^6^A writers and erasers on HIV-1 gene expression. As such, it was reported that knockdown of METTL3 and METTL14 resulted in reduced levels of CAp24 and/or Gag proteins in cells and supernatants and reduced levels of total levels of gp120 mRNA while knockdown of FTO and/or ALKBH5 have the opposite effects (Lichinchi et al., 2016a; Tirumuru et al., 2016). However, it has been always assumed that the effects on HIV-1 gene expression observed upon knockdown of m^6^A writers and erasers are a consequence of the hypomethylation and hypermethylation of the viral RNAs, respectively. In this regard, it would be important to evaluate the methylation status of the HIV-1 gRNA while performing these knockdown experiments. In addition, it would also be of interest to determine whether the role of FTO on HIV-1 gene expression is exerted on m^6^A or m^6^Am. Still, it is also possible that depletion of these RNA demethylases results in an indirect effect in the HIV-1 replication cycle. In this regard and considering that the reversibility of adenosine methylation was recently challenged (Darnell et al., 2017; Ke et al., 2017; Rosa-Mercado et al., 2017), it is very important to determine whether HIV-1 transcripts are indeed hypermethylated in ALKBH5 and FTO knockdown cells.

At the molecular level, m^6^A was first proposed to play a role in Rev-mediated nuclear export using the Rev-dependent gp120 mRNA as a model. However, from the data presented by Lichinchi et al. (2016a), it seems that the impact of m^6^A in mRNA abundance is stronger than the impact observed on relative nuclear export. A tempting explanation for this could be that reduced binding of Rev to the gp120 mRNA in METTL3/METTL14 knockdown cells also results in a diminished stability. In this sense, hypomethylation of the RRE may reduce the affinity of Rev leading to nuclear retention and degradation of Rev-dependent transcripts similar to what has been reported for proviruses lacking Rev (Chang and Sharp, 1989; Felber et al., 1989). Therefore, the increase in CAp24 and Gag proteins in cells and supernatants as well as in total levels of gp120 mRNA observed in ALKBH5 and/or FTO silenced cells could be explained by a higher affinity of Rev for hypermethylated viral transcripts that are efficiently exported to the cytoplasm and translated. Whether the effect of m^6^A on Rev-mediated nuclear export is conserved in all HIV-1 Rev-dependent transcripts (singly spliced and unspliced transcripts) or whether knockdown of m^6^A writers and erasers impact on gene expression from multiply spliced, Rev-independent, transcripts has not been evaluated.

**FIGURE 3:**
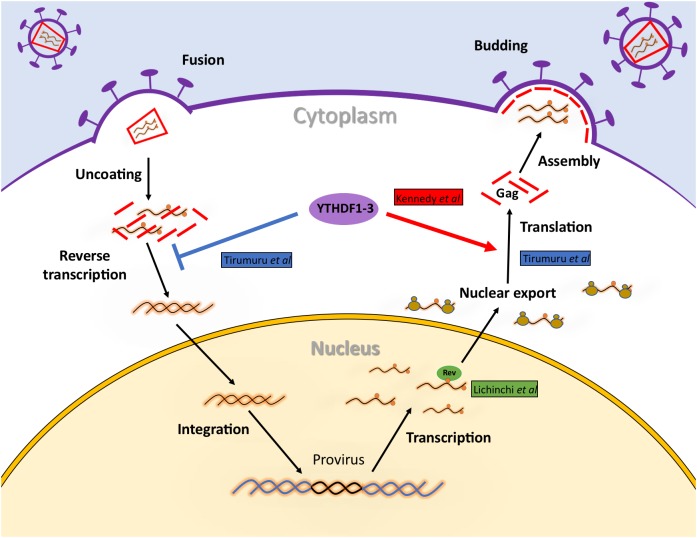
Regulation of HIV-1 RNA metabolism by m^6^A and the m^6^A machinery during viral replication. Early upon entry, m^6^A reader proteins YTHDF1, 2, and 3 induce the degradation of the incoming HIV-1 genomic RNA impeding reverse transcription and the subsequent steps of viral replication. However, once integrated, the presence of m^6^A within the RRE will favor nuclear export by facilitating Rev binding. In addition, binding of YTHDF1, 2, and 3 to the 3′-UTR of viral transcripts will increase their abundance and ribosome association resulting in increased levels of proteins synthesis.

Despite the role of m^6^A writers and erasers on viral gene expression from Rev-dependent transcripts (gag and gp120 mRNAs) seems clear, the role of cytoplasmic m^6^A readers is still controversial (**Figure 3**). This is probably due to differences in the methodologies employed in these studies. As such, data from Kennedy et al. (2016) were obtained at 24 or 48 h post-infection (hpi) using 293T cells stably overexpressing GFP (as a control) or one of the three YTHDF proteins. By using this system, they employed Western blot and RT-qPCR and show that YTHDF1, 2, and 3 promotes indistinctly HIV-1 Gag and Nef synthesis by enhancing the abundance of the cognate viral RNA. They also used a CRISPR/Cas9-mediated gene editing in order to generate an YTHDF2 knockout Jurkat cell line and further demonstrate the positive role of this m^6^A reader in viral gene expression. In contrast, Tirumuru et al. (2016) reported that the three cytoplasmic m^6^A readers induced the degradation of the incoming genomic RNA (which is expected to be identical to the gag mRNA). However, these experiments were performed both by transiently overexpressing or knocking down YTHDF1-3 proteins in HeLa cells infected with an HIV-1-Luc/VSV-G or by using HeLa/CD4+ infected with a wild type virus. Moreover, authors instead focused in the early steps of viral replication by quantifying reverse transcription (RT) products by qPCR. They also extended these observations to infection in primary T-cells, which is a more physiological model. Thus, it was reported that the early degradation of the viral genome that enters the cell results in the reduced accumulation of reverse transcription products, which leads to the concomitant inhibition of viral replication (Tirumuru et al., 2016). More recently, this group showed that viruses produced from 293T cells transiently overexpressing YTHDF1-3 proteins have a reduced infectivity while viruses from YTHDF1 or YTHDF3 knockdown cells are more infective (Lu et al., unpublished). Although this methodology was similar to that employed by Kennedy et al. (2016) reported that both overexpression and knockdown of the m^6^A readers results in reduced intracellular levels of Gag thus, suggesting that balanced levels of YTHDF1-3 proteins are required for proper Gag synthesis. Unfortunately, the levels of intracellular gRNA under overexpression and knockdown conditions were not evaluated and thus, it is not possible to determine whether the effects of YTHDF1-3 proteins on Gag synthesis observed by Lu et al. (unpublished) were exerted at the level of the gRNA. Instead, authors proposed that the cytoplasmic m^6^A readers might regulate the intracellular processing of Gag. Although the molecular mechanisms by which YTHDFs are able to enhance or reduce HIV-1 gRNA and Gag levels are still unknown, these opposite observations reported by the Cullen and Wu groups are interesting in several ways.

The first interesting observation is that both studies reported a redundant role for these three m^6^A readers. This is striking since despite their high homology, YTHDF proteins were shown to play different roles in cellular mRNA metabolism (Meyer and Jaffrey, 2017; Patil et al., 2017). YTHDF1 was shown to interact with translation initiation factor eIF3 and promote translation initiation of its target mRNAs (Wang et al., 2015). On the other hand, YTHDF2 was shown to reduce the half-life of its mRNA targets (Wang et al., 2014; Du et al., 2016). YTHDF3 was shown to associate with YTHDF1 and YTHDF2 promoting mRNA translation and decay, respectively (Shi et al., 2017). Interestingly, the dichotomy of the roles of YTHDFs proteins has also been observed in other viruses. As such, the cytoplasmic m^6^A readers were shown to stimulate Influenza virus and SV40 replication but to inhibit Hepatitis C, Zika virus and KSHV replication (Gokhale et al., 2016; Lichinchi et al., 2016b; Courtney et al., 2017; Tan et al., 2017; Tsai et al., 2018). Although the ability of YTHDF1-3 proteins to promote or inhibit viral replication could be explained by their ability to interact with members of the translation and mRNA decay machineries (Wang et al., 2014; Du et al., 2016; Shi et al., 2017), the molecular mechanisms at play are still unknown and further work is necessary to better understand the function of the cytoplasmic m^6^A readers during viral replication. In this regard, the effects of YTHDF1-3 on HCV replication have already provided useful insights into the mechanism of action of these host proteins. Indeed, it was shown that YTHDF1-3 proteins were enriched in lipids droplets, which correspond to the sites where viral particle assembly take place (Gokhale et al., 2016). Interestingly, the authors showed that YTHDF2 has reduced affinity for an HCV mutant lacking m^6^A sites at the E1 region of the viral genome while the viral Core protein was preferentially bound to the same viral RNA mutant. Since the HCV Core protein drives viral particle assembly at lipid droplets, the authors proposed that YTHDF1-3 proteins inhibit viral replication by interfering with viral RNA packaging mediated by Core (Gokhale et al., 2016). For HIV-1, this idea is supported by data showing that YTHDF1-3 bind to methylated residues at the 5′-UTR of the gRNA as well as by data showing that YTHDF2 interacts with the Gag protein in an RNA-dependent manner (Tirumuru et al., 2016; Lu et al., unpublished). Thus, it would be of great interest to investigate whether the binding of cytoplasmic m^6^A readers to the HIV-1 5′-UTR interferes with the binding of Gag and gRNA packaging.

Another interesting observation came from the YTHDF1-3 CLIP-seq data reported by Tirumuru et al. (2016) and Kennedy et al. (2016) which shows that only some of the binding sites identified along the HIV-1 genome overlap with m^6^A sites (**Figure 2**). This observation led to the possibility that YTHDF proteins bind the viral RNA in m^6^A-dependent and independent manners or that they are associated to the viral genome indirectly through additional RNA-binding proteins. In this regard, it is completely unknown whether the redundant role of YTHDF1, 2, and 3 on HIV-1 gene expression depends or not on the presence of m^6^A along the viral RNA. Thus, it would be of interest to evaluate the impact of YTHDFs proteins under conditions in which viral transcripts are either hypermethylated or hypomethylated. As mentioned above, it is also unknown which is the molecular mechanism by which YTHDF1, 2, and 3 overexpression results in increased levels of HIV-1 transcripts. Still, it seems that binding of YTHDF proteins to the 3′-UTR of any target mRNAs is sufficient to exert a positive effect on abundance (Kennedy et al., 2016). Since YTHDF1-3 are cytoplasmic proteins (Meyer and Jaffrey, 2017; Patil et al., 2017), one could expect that these m^6^A readers act by stabilizing the mRNA at this subcellular compartment. Thus, it is possible that the recruitment of YTHDF1, 2, or 3 to the HIV-1 mRNAs could either interfere with the recruitment of an RNA destabilizing factor or promote the recruitment of any RNA stabilizing protein. Additionally, the fact that increased gag and nef mRNA levels were accompanied by an increase in Gag and Nef proteins (Kennedy et al., 2016), suggest that YTHDF-bound viral transcripts are substrates for the cellular translational machinery. Interestingly, YTHDF1 and YTHDF3 were shown to share several protein partners, most of them related to the translational machinery (Shi et al., 2017). Thus, it would be of interest to evaluate which of these common partners are also shared by YTHDF2 in order to identify a common mechanism by which cytoplasmic m^6^A readers exert positive yet redundant functions on HIV-1 mRNA abundance and protein synthesis.

The role of YTHDF1, 2, and 3 on HIV-1 genome degradation early during infection is a bit more difficult to explain. Several reports have shown that reverse transcription begins within the viral particle (Lori et al., 1992; Trono, 1992; Zhang et al., 1998) and thus, one could expect that YTHDF proteins should be packaged to induce the degradation of the viral genome upon entry into the cell. In addition, it is still unclear when and where uncoating takes place (Campbell and Hope, 2015). If most of the uncoating occurs at the nuclear pore complex as it has been proposed (Arhel et al., 2007; Campbell and Hope, 2015), it seems difficult that cytoplasmic m^6^A readers encounter the HIV-1 genome in the cytoplasm to induce degradation.

The m^6^A-mediated regulation of HIV-1 RNA metabolism and replication is a fascinating emerging field that added a new layer of complexity to the already complex regulation of HIV-1 gene expression. Still, there are several questions that remain unanswered. For instance, despite components of the m^6^A machinery have been shown to modulate the abundance of gag, gp120, nef, tat, and rev mRNAs, m^6^A sites have been only mapped to the HIV-1 genomic RNA and, to date, it is unclear whether these methylations are indeed present in singly and multiply spliced transcripts. Moreover, it is also unknown where within the cell viral transcripts encounter m^6^A writers. Indeed, the m^6^A writer co-factor WTAP was proposed to drive the localization of the METTL3/METTL14 complex to nuclear speckles, sites enriched in pre-mRNA splicing factors (Ping et al., 2014). Although methylation at nuclear speckles would be possible for singly spliced transcripts (which are expected to localize in nuclear speckles during splicing), multiply spliced transcripts and the unspliced RNA genome are not expected to encounter the m^6^A writer complex at these sites within the nucleus. However, m^6^A was recently shown to be deposited co-transcriptionally (Ke et al., 2017; Slobodin et al., 2017), suggesting that the 9-kb RNA could be methylated prior it undergoes alternative splicing to generate multiply spliced and singly spliced transcripts. In this regard, all viral transcripts would be expected to carry the m^6^A modification independently of their splicing status.

Given the fact that the presence of m^6^A also alters RNA structures (Liu et al., 2015, 2017; Roost et al., 2015; Spitale et al., 2015), it would be great of interest to determine whether this modification affects structures along the gRNA, especially those located within the 5′-UTR, which is composed by several RNA structural motifs involved in different steps of the replication cycle or the RRE, which was already shown to be involved in the m^6^A-mediated regulation of viral gene expression.

Additional aspects to be addressed are related to the role of other m^6^A reader proteins. While the role of the nuclear reader YTHDC1 on HIV-1 gene expression has not been investigated, a large-scale siRNA screen identified YTHDC2 as a host factor required for HIV-1 replication (Brass et al., 2008). However, whether YTHDC1 and 2 play a role in HIV-1 replication needs to be evaluated.

A recent report showed that the presence of m^6^A results in the repulsion of some proteins such as the stress granules dependency factors G3BP1 and G3BP2 (Edupuganti et al., 2017). Interestingly, binding of G3BP1 to the HIV-1 genomic RNA inhibits viral replication in T-cells and macrophages (Cobos Jiménez et al., 2015). Thus, an additional function of m^6^A would be to repeal the binding of inhibitory proteins such as G3BP1 in order to ensure efficient translation and packaging. In this regard, it would be of great interest to evaluate the proteome bound to the HIV-1 genomic RNA in the presence or absence of m^6^A.

Last but not least, the methylation inhibitor 3-deazaadenosine (DAA) was reported as an inhibitor of viral gene expression and replication (Flexner et al., 1992; Gordon et al., 2003; Kennedy et al., 2016), indicating that viral RNA methylation should also be considered as a target for the development of novel antiretroviral drugs.

## Author Contributions

SR-B, CP-M, FV-E, and RS-R participated in the writing and editing of the manuscript. SR-B drew the figures. All authors approved the final version.

## Conflict of Interest Statement

The authors declare that the research was conducted in the absence of any commercial or financial relationships that could be construed as a potential conflict of interest.
